# The impact of postoperative radiotherapy on the survival of patients with stage III non-small cell lung cancer: A CONSORT-compliant analysis using the SEER database

**DOI:** 10.1097/MD.0000000000034015

**Published:** 2023-06-16

**Authors:** Kun Wu, Wang Peng, Zhifeng Shuai, Xin Peng, Haibo Liu, Sanhong Zhang

**Affiliations:** a Department of Cardiothoracic Surgery, Xiangtan Central Hospital, Xiangtan, Hunan, China; b School of Medical Equipment and Management, Xiangtan Medicine and Health Vocational College, Xiangtan, Hunan, China; c Department of Radiology, Xiangtan Central Hospital, Xiangtan, Hunan, China; d Department of Radiology, Liuyang Chinese Medicine Hospital, Changsha, Hunan, China.

**Keywords:** and end results (SEER) database, epidemiology, postoperative radiotherapy, stage III non-small cell lung cancer, surveillance, survival benefit

## Abstract

Postoperative radiotherapy (PORT) is commonly used to treat patients with resected stage III non-small cell lung cancer (NSCLC), but its effectiveness remains uncertain. This retrospective cohort study aimed to investigate the impact of PORT on overall survival (OS) and evaluate its heterogeneity among subgroups of patients.

**Methods:**

A total of 6305 patients with resected stage III NSCLC were included in this study from the Surveillance, Epidemiology, and End Results (SEER) database. Propensity score matching was conducted to balance baseline characteristics between the patients who received PORT and those who did not. OS was used as the primary outcome. Subgroup analysis was performed to identify which patient subgroups might benefit more from PORT.

**Results:**

Overall, no significant difference was observed in OS between the 2 groups with or without propensity score matching. However, subgroup analysis demonstrated that PORT improved OS in patients with certain characteristics, including stage IIIA/N2, stage IIIB, squamous cell carcinoma, tumor grade III-IV, or lymph node ratio (LNR) > 1/3. Multivariate analysis showed that several variables were associated with adverse prognostic factors for OS, such as marital status (others), race (white), male gender, squamous cell carcinoma, elderly age, advanced stage, poor histological differentiation grade, high LNR, and not receiving chemotherapy.

**Conclusion:**

In patients with resected stage III NSCLC, PORT may not be beneficial for all patients. However, it may improve survival time in certain patient subgroups, such as those with stage IIIA/N2, stage IIIB, squamous cell carcinoma, tumor grade III to IV, or LNR > 1/3. These findings provide important information for clinical decision-making and future research regarding the use of PORT in patients with resected stage III NSCLC.

## 1. Introduction

Lung cancer is a leading cause of cancer mortality. Approximately 85% of lung cancers are non-small cell lung cancer (NSCLC), and 1-third of these individuals have stage III NSCLC, a complex and challenging disease with high heterogeneity and poor prognosis that requires effective multimodality therapy.^[1,2]^

Radiotherapy is an important treatment modality for lung cancer, as locoregional recurrence is linked to poor overall survival (OS). Postoperative radiation therapy (PORT) is frequently indicated to enhance local tumor control in NSCLC patients with stage III/N+. However, the utility of PORT for patients with resected stage III NSCLC remains debatable. Previous studies have shown that PORT has a negative effect on patients with totally resected stage I to III NSCLC.^[3–5]^ However, the role of radiotherapy may be underestimated in stage III NSCLC due to the low-risk of local recurrence in patients with pN0-1 and the relatively high toxicity of outdated radiotherapy techniques. A randomized study showed a positive effect of PORT for pN2 NSCLC patients and pN1 NSCLC patients without chemotherapy.^[6]^ Studies have also revealed that PORT could improve outcomes for entirely resected stage III/N2 NSCLC patients.^[7–10]^ Because of the varying quality of studies and differences in clinical outcome selection, there is insufficient evidence and considerable uncertainty regarding the efficacy of PORT for stage III NSCLC treatment. Thus, a randomized controlled trial (RCT) is necessary to assess the role of contemporary PORT in postsurgical NSCLC patients. Recent trials have demonstrated that PORT could decrease locoregional recurrence but has no effect on survival, so it should not be regarded as a standard care for patients with resected stage IIIA/N2 NSCLC.^[11,12]^ However, arbitrarily denying the benefit of PORT based solely on statistical findings of the RCT stated above is not ideal. After the presentation of the Lung ART trial, 82 percent of European radiation oncology specialists in lung cancer continue to utilized PORT for pN2 NSCLC patients with factors of risk.^[13]^ Thus, further analysis is needed to determine which patient subgroups would benefit most from PORT. Unfortunately, no established clinical or biomedical signs exist to choose potential candidates for PORT at this time.

Given the highly unpredictable clinical outcomes of stage III NSCLC due to its heterogeneous nature, this study employed propensity score matching (PSM) to mitigate significant selection bias between 2 groups and facilitate direct comparison of specific interventions. The most current national data from the Surveillance, Epidemiology, and End Results (SEER)-Medicare database was utilized to evaluate the association between PORT and prolonged survival in resectable stage IIIA and IIIB NSCLC patients, while identifying the patient subgroups that might derive the most optimal benefits from PORT treatment.

## 2. Methods

### 2.1. Data sources

The patient information was collected from the SEER database which covered almost 28% of the US population and included data from 18 population-based registries between 2000 and 2019. We used SEER*Stat software (v8.3.6, https://seer.cancer.gov/seerstat/) to enroll patients for this study. As the data was obtained from a public database and all identifying information has been removed, ethical approval was not required. Additionally, the need for informed consent was waived due to the deidentified nature of the data used in this study.

### 2.2. Patient population

We retrieved data on patients diagnosed with American Joint Committee on Cancer stage III lung cancer from the SEER database (n = 48,268). We excluded patients who did not undergo surgery (n = 39,287), those classified as stage IIIC (n = 104), and those with an unclear pathological diagnosis (n = 2572).

**Figure 1.**
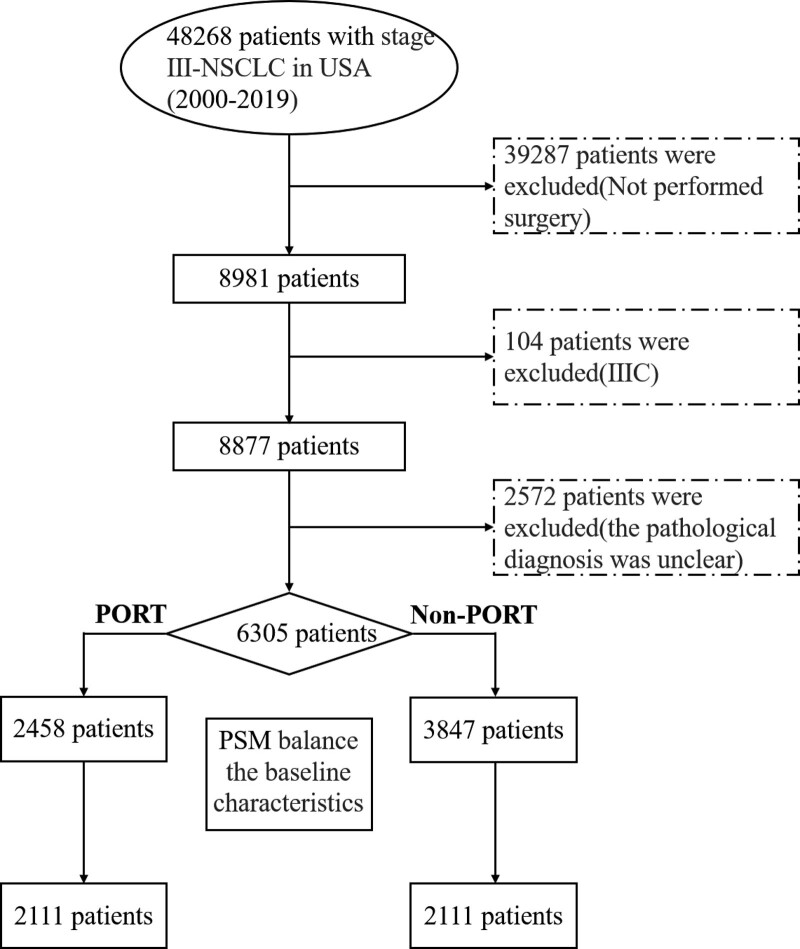
Flowchart illustrating the selection of the study population.

As a result of the inclusion criteria (Fig. 1), a total of 6305 patients were incorporated into our study for analysis. The median follow-up time for all patients still alive was 82 months.

The following patient’s characteristics were collected: stage (IIIA, IIIB), age at diagnosis (≤ 60 years old, > 60 years old), the marital status (married, others), sex (male, female), race (white, black, others), the tumor grade (grade I, grade II, grade III–IV), the primary site (lower lobe, middle lobe, lower lobe), laterality (left, right), tumor histology (adenocarcinoma, squamous cell carcinoma, others), T stage (T0–1, T2, T3, T4), N stage (N0, N1, N2, N3), extent of surgery (sub-lobectomy, lobectomy/bilobectomy), a positive to sampled lymph node ratio (LNR) (> 1/3, ≤ 1/3), PORT (no, yes), chemotherapy (no/unknown, yes) and survival time (from diagnosis to death or last follow-up).

### 2.3. Statistical analysis

We performed all statistical analyses using R version 3.6.3 software (https://www.r-project.org/) with the following packages: table 1, survey, reshape2, MatchIt, survival, and survminer. Categorical variables were presented as numbers and percentages (N, %), and we used the Pearson chi-square test to compare differences between these variables.

To balance patient assignment in our analysis of the effect of PORT on resected stage III NSCLC, we employed PSM using nearest-neighbor matching with a caliper value of 0.05. We then utilized the Kaplan–Meier method and log-rank test to compare OS between the PORT and non-PORT groups, as well as calculate the median survival time. Additionally, we conducted univariate and multivariate Cox regression analyses to explore prognostic factors affecting OS in patients with resected stage III NSCLC.

## 3. Results

### 3.1. Patient characteristics

**Figure 2.**
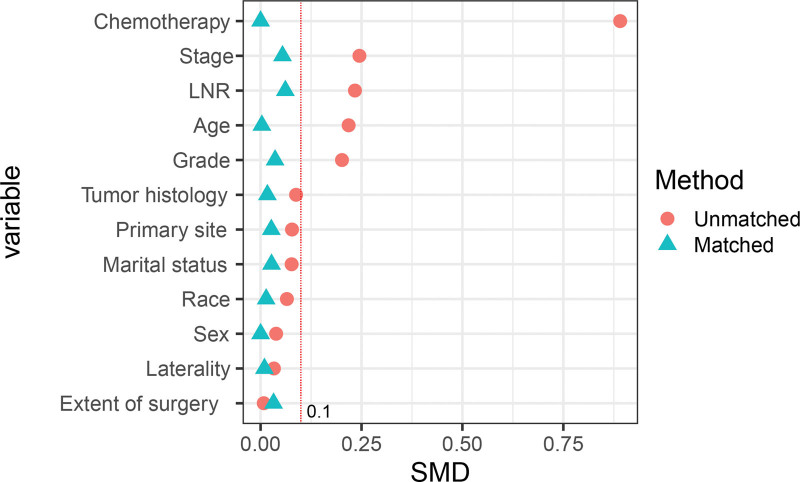
Visualization of propensity score matching to balance baseline characteristics. The standardized mean difference (SMD) of each variable is <0.1 after propensity score matching. PSM = propensity score matching, SMD = standardized mean difference.

In our study of 6305 patients with resected stage III NSCLC, 2458 (38.98%) received PORT. Table 1 shows that several clinicopathological characteristics were significantly more common in the PORT group, including married status, white race, elderly male patients with lower lobe tumors, advanced stage disease, adenocarcinoma histology, high LNR, and poor tumor grade (*P* < .05). After applying PSM, all variables were well-balanced between the PORT (n = 2111) and non-PORT (n = 2111) groups, as demonstrated in Table 1 and Figure 2.

**Table 1:** Comparison of patient characteristics before and after propensity score matching in the study of postoperative radiation therapy for resected stage III non-small cell lung cancer.

Characteristics	Level	Unmatched			Matched		
		Non-PORT	PORT	*P* value	Non-PORT	PORT	*P* value
n		3847	2458		2111	2111	
Marital status (%)	Others	1624 (42.2)	945 (38.4)	.003	824 (39.0)	852 (40.4)	.396
	Married	2223 (57.8)	1513 (61.6)		1287 (61.0)	1259 (59.6)	
Age (%)	≤60 yr old	855 (22.2)	783 (31.9)	<.001	611 (28.9)	614 (29.1)	.946
	>60 yr old	2992 (77.8)	1675 (68.1)		1500 (71.1)	1497 (70.9)	
Race (%)	White	3243 (84.3)	2012 (81.9)	.004	1751 (82.9)	1740 (82.4)	.904
	Black	309 (8.0)	228 (9.3)		182 (8.6)	188 (8.9)	
	Others	295 (7.7)	218 (8.9)		178 (8.4)	183 (8.7)	
Sex (%)	Male	1934 (50.3)	1283 (52.2)	.143	1102 (52.2)	1102 (52.2)	1.000
	Female	1913 (49.7)	1175 (47.8)		1009 (47.8)	1009 (47.8)	
Tumor grade (%)	Grade I	380 (9.9)	128 (5.2)	<.001	111 (5.3)	126 (6.0)	.506
	Grade II	1646 (42.8)	992 (40.4)		885 (41.9)	897 (42.5)	
	Grade III/IV	1821 (47.3)	1338 (54.4)		1115 (52.8)	1088 (51.5)	
Primary Site (%)	Upper lobe	1288 (33.5)	734 (29.9)	.011	656 (31.1)	664 (31.5)	.689
	Middle lobe	189 (4.9)	127 (5.2)		102 (4.8)	113 (5.4)	
	Lower lobe	2370 (61.6)	1597 (65.0)		1353 (64.1)	1334 (63.2)	
Laterality (%)	Left	1711 (44.5)	1053 (42.8)	.211	923 (43.7)	913 (43.2)	.780
	Right	2136 (55.5)	1405 (57.2)		1188 (56.3)	1198 (56.8)	
Stage (%)	III A	3301 (85.8)	1875 (76.3)	<.001	1732 (82.0)	1687 (79.9)	.084
	III B	546 (14.2)	583 (23.7)		379 (18.0)	424 (20.1)	
Tumor histology (%)	Adenocarcinoma	1666 (43.3)	1131 (46.0)	.003	952 (45.1)	935 (44.3)	.861
	Squamous cell carcinoma	865 (22.5)	587 (23.9)		500 (23.7)	511 (24.2)	
	Others	1316 (34.2)	740 (30.1)		659 (31.2)	665 (31.5)	
LNR (%)	≤1/3	2926 (76.1)	1610 (65.5)	<.001	1518 (71.9)	1459 (69.1)	.050
	>1/3	921 (23.9)	848 (34.5)		593 (28.1)	652 (30.9)	
Extent of surgery	Sub-lobectomy	451 (11.7)	282 (11.5) 0.793		216 (10.2)	1874 (88.8) 0.217	
	Lobectomy/bilobectomy	3396 (88.3)	2176 (88.5)		1895 (89.8)	250 (11.8)	
Chemotherapy (%)	No/unknown	1805 (46.9)	250 (10.2)	<.001	250 (11.8)	250 (11.8)	1.000
	Yes	2042 (53.1)	2208 (89.8)		1861 (88.2)	1861 (88.2)	

LNR = a positive to sampled lymph node ratio, PORT = postoperative radiotherapy.

**Figure 3.**
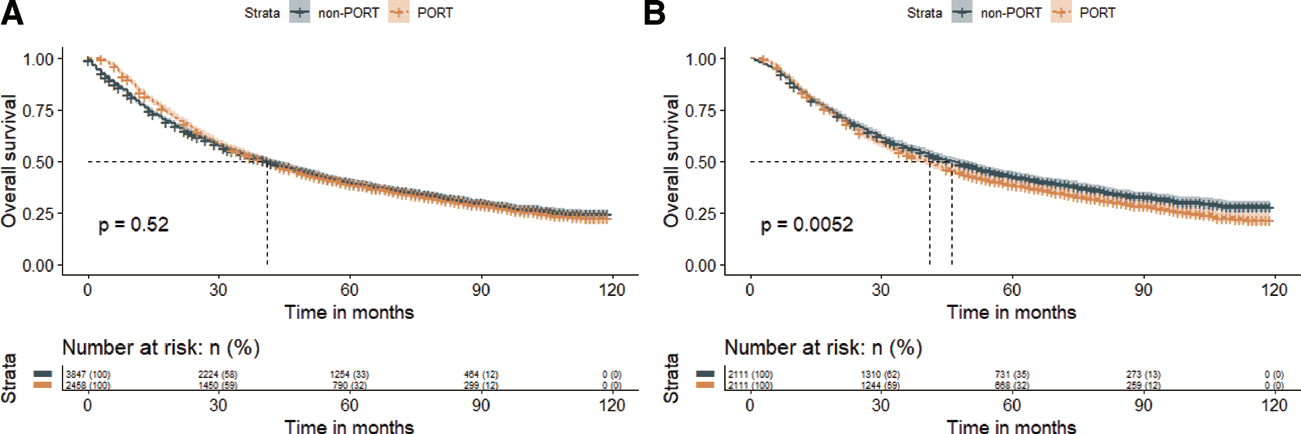
Kaplan–Meier curves demonstrating overall survival in patients who received PORT or did not receive PORT before and after propensity score matching. Panel A displays the curves before propensity score matching, while panel B displays the curves after propensity score matching. PORT = postoperative radiation therapy.

Before PSM, there was no significant difference in OS between the PORT and non-PORT groups (median survival time [MST]: 41 months vs 41 months; *P* = .520). However, after PSM, the MST of OS was significantly worse in the PORT group compared to the non-PORT group (41 months vs 46 months; *P* = .005), as shown in Figure 3.

### 3.2. Subgroup analysis

The result of subgroup analysis was summarized in Table 2. Based on the heterogeneity of the PORT effect among subgroups, we analyzed whether a difference in survival outcomes existed between the PORT and the non-PORT groups according to tumor stage, tumor grade, tumor histology, and LNR.

**Table 2:** Assessment of heterogeneity in the effect of postoperative radiation therapy among subgroups in patients with resected stage III non-small cell lung cancer.

Subgroup	n = 6305	MST (months)		*P* value
		non-PORT	PORT	
Stage				
III A/N0–1	2208	48	36	.010
III A/N2	2968	41	45	.018
III B	1129	27	32	.005
Age				
≤60 yr old	1638	62	55	.270
>60 yr old	4667	37	37	.970
Race				
White	5255	39	39	.420
Black	537	45	47	.700
Others	513	56	51	.270
Sex				
Male	3217	31	34	.150
Female	3088	53	50	.840
Primary site				
Upper lobe	2022	37	35	.520
Middle lobe	316	56	40	.320
Lower lobe	3967	42	43	.190
Laterality				
Left	2764	42	41	.370
Right	3541	41	41	.930
Tumor grade				
Grade I–II	3146	50	41	.025
Grade III–IV	3159	31	38	<.001
Tumor histology				
Adenocarcinoma	2797	44	44	.410
Squamous cell carcinoma	1452	30	32	.043
Others	2056	46	41	.150
LNR				
>1/3	1769	25	32	<.001
≤1/3	4536	50	46	.690
Extent of surgery				
Sub-lobectomy	733	45	33	.026
Lobectomy/bilobectomy	5572	41	41	.140
Chemotherapy				
No/unknown	2055	30	28	.980
Yes	4250	52	42	<.001

LNR = a positive to sampled lymph node ratio, MST = Median survival time, PORT = postoperative radiotherapy.

**Figure 4.**
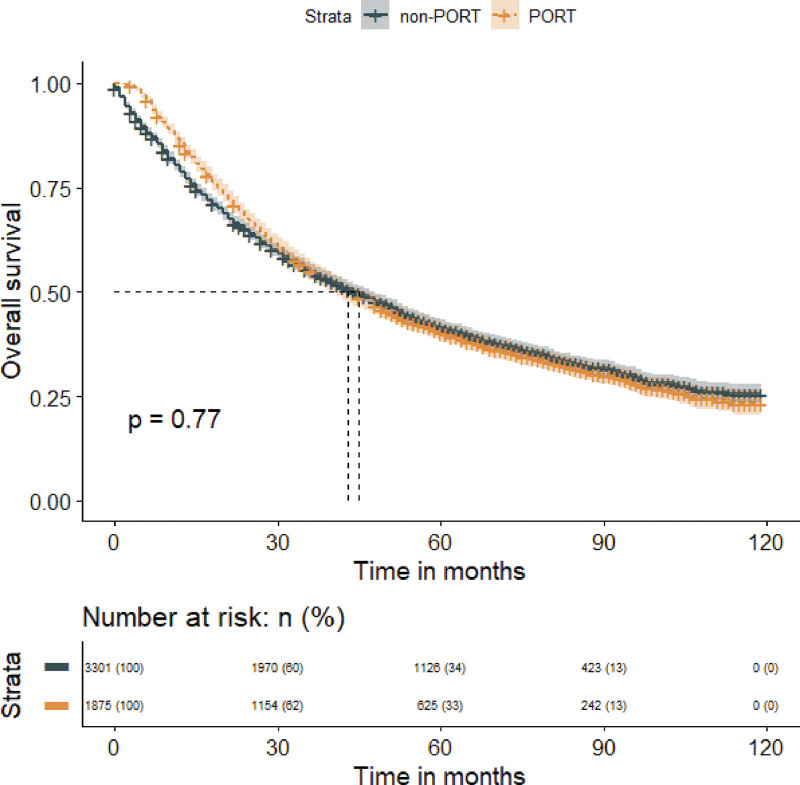
Overall survival in patients with stage IIIA non-small cell lung cancer who received PORT or did not receive PORT. PORT = postoperative radiation therapy.

**Figure 5.**
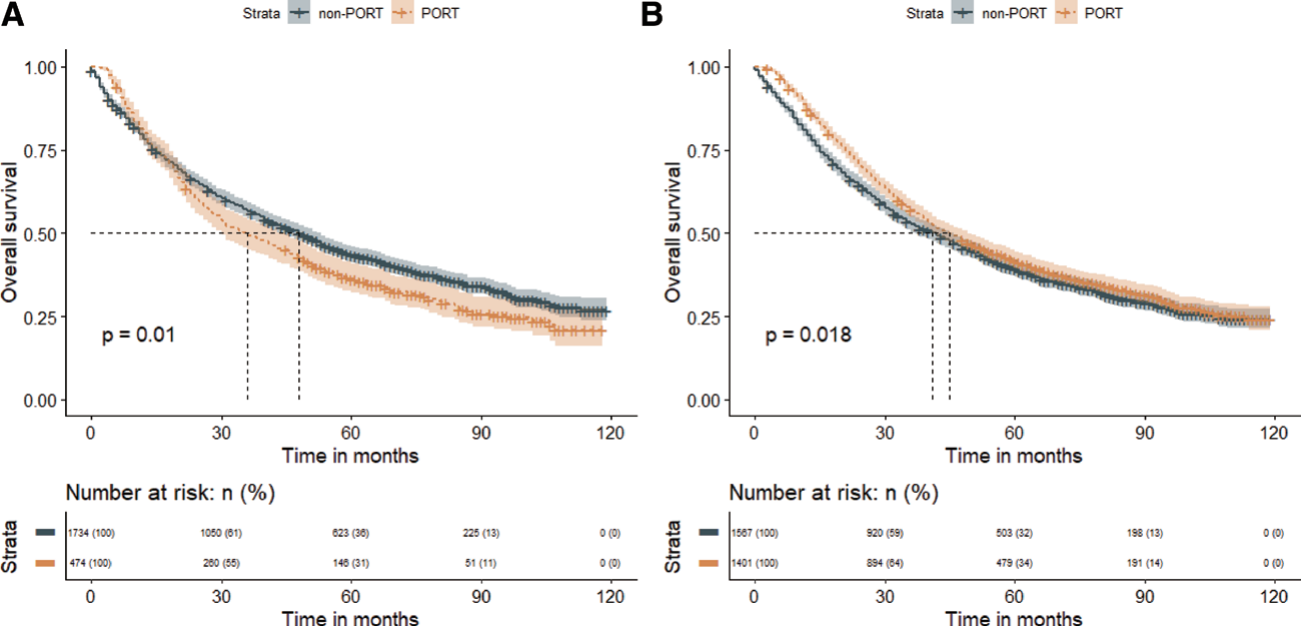
Overall survival in patients with stage IIIA/N0-1 (panel A) and stage IIIA/N2 (panel B) non-small cell lung cancer who received PORT or did not receive PORT. PORT = postoperative radiation therapy.

**Figure 6.**
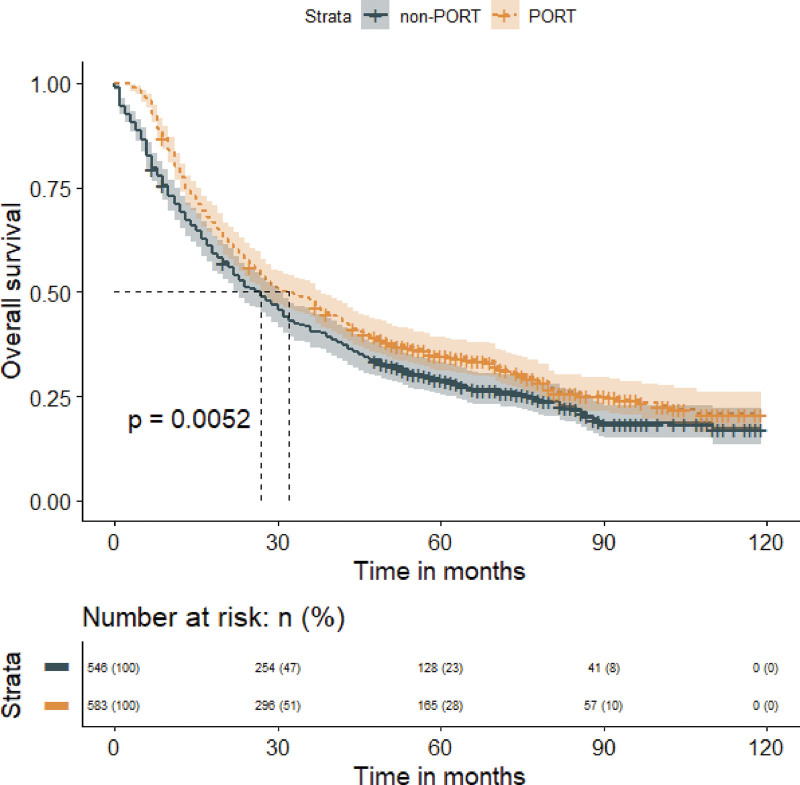
Overall survival in patients with stage IIIB non-small cell lung cancer who received PORT or did not receive PORT. PORT = postoperative radiation therapy.

Our study found no significant difference in MST between patients with stage IIIA NSCLC who received postoperative radiotherapy (PORT) versus those who did not (43 months vs 45 months; *P* = .770), as shown in Figure 4. However, PORT was associated with a statistically significant prolongation of MST in patients with stage IIIA/N2 NSCLC (45 months vs 41 months; *P* = .018), but a worsened MST in patients with stage IIIA/N0-1 NSCLC (36 months vs 48 months; *P* = .010), as indicated in Figure 5. Additionally, our analysis demonstrated that PORT significantly prolonged the MST in patients with stage IIIB NSCLC compared to non-PORT patients (32 months vs 27 months; *P* = .005), as shown in Figure 6.

**Figure 7.**
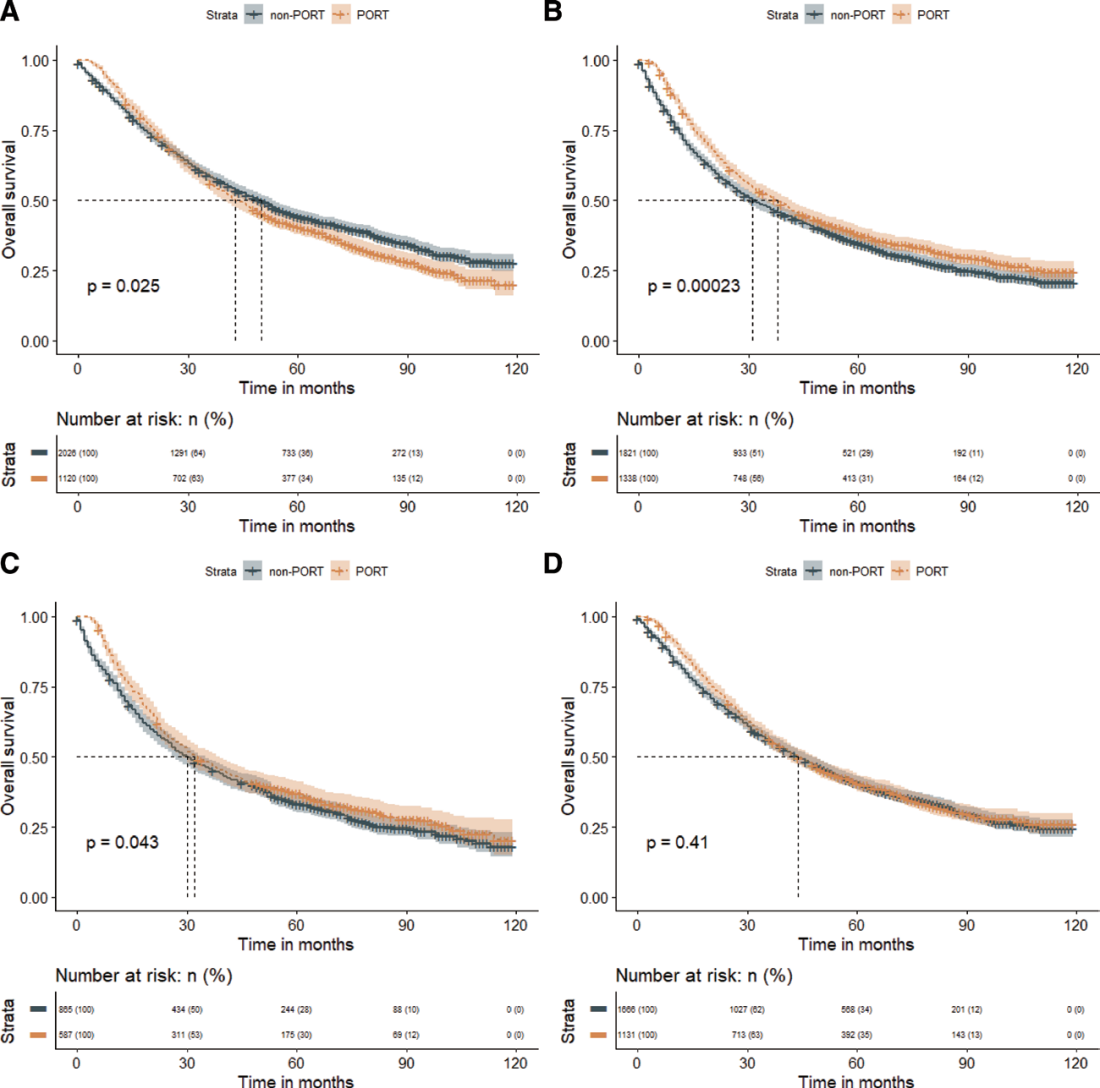
Subgroup analysis of tumor grade or histology demonstrating overall survival in patients who received PORT or did not receive PORT. Panel A represents the subgroup of tumor grade I–II, panel B represents the subgroup of tumor grade III–IV, panel C represents the subgroup of squamous cell carcinoma, and panel D represents the subgroup of adenocarcinoma. PORT = postoperative radiation therapy.

**Figure 8.**
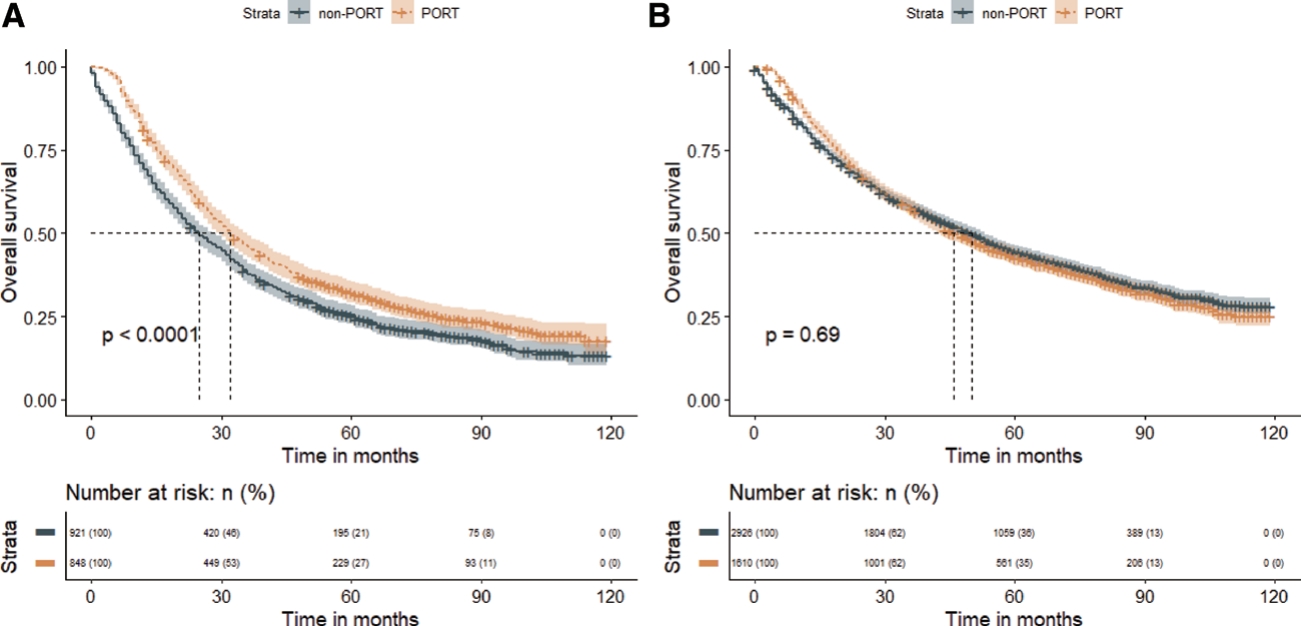
Overall survival in patients who received PORT or did not receive PORT postoperative based on LNR. PORT = postoperative radiation therapy, LNR = positive to sampled lymph node ratio.

In the subgroup analysis of the tumor grade, PORT significantly prolonged MST in patients with grade III-IV NSCLC (PORT vs non-PORT: 38 months vs 31 months; *P* < .001), but resulted in a significantly worse MST in patients with grade I-II NSCLC (PORT vs non-PORT: 41 months vs 50 months; *P* = .025). Similarly, in the subgroup analysis of the tumor histology, PORT did benefit the patient with squamous cell carcinoma (MST, PORT vs non-PORT: 32 months vs 30 months; *P* = .043), but not the adenocarcinoma (MST, PORT vs non-PORT: 44 months vs 44 months; *P *= .410) (Fig. 7).Finally, we discovered that PORT improved OS in a subset of patients with the LNR > 1/3 (MST, PORT vs non-PORT: 32 months vs 25 months; *P* < .001), but not in patients with the LNR ≤ 1/3 (MST, PORT vs non-PORT: 46 months vs50 months; *P* = .690) (Fig. 8).

### 3.3. Prognostic factors

Our study utilized rigorous univariate and multivariate Cox regression analysis to unveil a set of prognostic factors that significantly impacted OS in resected stage III NSCLC patients. Results indicated that several clinical and pathological characteristics were potential adverse prognostic factors for OS, including marital status (others), white race, male gender, squamous cell carcinoma histology, elderly age, advanced disease stage, poor histological differentiation grade, LNR, and not receiving chemotherapy (*P* < .001). A comprehensive description of these findings, complete with detailed hazard ratios, 95% confidence intervals, and corresponding p-values, are presented in Table 3.

**Table 3:** Results of Cox proportional-hazards regression model analysis for overall survival in patients with resected stage III non-small cell lung cancer who received or did not receive postoperative radiation therapy.

Characteristics	Univariate	*P* value	Multivariate	*P* value
	HR (95% CI)		HR (95% CI)	
Marital status (vs others)	0.93 (0.87–0.99)	.016	0.88 (0.83–0.94)	<.001
Age (vs≤60 yr old)	1.49 (1.38–1.6)	<.001	1.39 (1.29–1.50)	<.001
Race (vs white)				
Black	0.92 (0.82–1.03)	.137	0.93 (0.83–1.04)	.198
Others	0.80 (0.71–0.9)	<.001	0.82 (0.73–0.92)	.001
Sex (vs male)	0.72 (0.68–0.76)	<.001	0.71 (0.67–0.76)	<.001
Primary site (vs Lower lobe)				
Middle lobe	0.8 (0.69–0.93)	.100		
Lower lobe	0.88 (0.82–0.94)	.100		
Laterality (vs left)	1.02 (0.96–1.08)	.583		
Stage (vs IIIA)	1.31 (1.21–1.41)	<.001	1.30 (1.21–1.41)	<.001
Grade (vs grade I)				
Grade II	1.42 (1.25–1.61)	<.001	1.43 (1.26–1.63)	<.001
Grade III/IV	1.65 (1.46–1.88)	<.001	1.65 (1.45–1.89)	<.001
Extent of surgery (vs Sub-lobectomy)	1.01 (0.92–1.11)	.902		.231
Tumor histology (vs adenocarcinoma)				
Squamous cell carcinoma	1.23 (1.14–1.33)	<.001	1.13 (1.04–1.22)	.003
Others	1.02 (0.95–1.1)	.547	1.01 (0.94–1.08)	.775
LNR (vs≤1/3)	1.5 (1.4–1.6)	<.001		
Chemotherapy (vs No/unknow)	0.72 (0.67–0.76)	<.001	0.67 (0.62–0.71)	<.001
PORT (vs No)	0.98 (0.92–1.04)	.529		

LNR = a positive to sampled lymph node ratio, OS = Overall survival, PORT = postoperative radiotherapy.

## 4. Discussion

To our best knowledge, this is the latest large population-based cohort study in resected stage III NSCLC patients using the most recent updated data from the SEER database. In the present study, we found that patients with resected stage III NSCLC who received PORT failed to improve OS and had a poorer outcome post-PSM. It was also confirmed by the study of Perry et al,^[14]^ a 37-patient Phase III research found that no difference in survival between the PORT and the non-PORT groups. In addition, it was proved by a clinical trial-based meta-analysis that the patient who in PORT group had a poorer 2-year survival rate than the entire patient group (48% vs 55%), indicating that PORT was not conducive to improving the OS.^[4]^ Another meta-analysis of randomized trials demonstrated that PORT even caused a decreased survival rate, as well as the cardiopulmonary toxicity.^[15]^ However, other researches hold different views. Bogart et al^[16]^ suggested that patients with resected stage III NSCLC should be considered to receive PORT. The reasons for the above controversy may be that the different proportions of different subgroups or the different clinicopathological characteristics of enrolled patients. PORT often fails to ameliorate the survival status of patients in the low-risk subset, that is because the benefit from PORT is outweighed by the PORT-induced toxicity in the low-risk subset. These results therefore suggested that stage III NSCLC therapy should be customized. Additional researches are needed to identify stage III NSCLC patients who may benefit most from PORT.

Our study conducted a comprehensive subgroup analysis, which convincingly showed that postoperative radiation therapy (PORT) had a positive impact on OS in NSCLC patients with resected stage IIIA/N2 and IIIB. This finding aligns well with numerous prior studies, including an impressive meta-analysis of 8928 stage III/N2 NSCLC patients across eleven research trials, which demonstrated a trend toward prolonged OS with PORT treatment.^[17]^ Additionally, Pang et al^[18]^ found that NSCLC patients with stage IIIA/N2 who received PORT exhibited significantly improved survival compared to their non-PORT treated counterparts. These collective findings underscore the distinct advantages of PORT for suitable NSCLC patient cohorts, offering definitive proof of its effectiveness and clinical application. In a retrospective study conducted by Corso et al,^[8]^ the impact of PORT was analyzed in NSCLC patients with N2 involvement. The results demonstrated that PORT could potentially increase survival rates among patients with N2 metastasis. Yuan et al^[19]^ reported an increase in 5-year overall survival rate from 54.1% to 65.7% with the use of PORT in NSCLC patients with N2 metastasis in only 1 N2 station. However, 2 recently published RCTs concluded that PORT should not be routinely recommended for completely resected IIIA/N2 NSCLC patients.^[11,20]^ The recent RCTs challenged the conclusions of many previous studies, including some with large sample sizes. The reason for the difference in findings between the real-world study and the RCTs is likely due to the rigorous design and strict control of the treatment process in the RCTs as compared to retrospective studies. Additionally, participants in the RCTs have access to advanced diagnostic tests such as PET/CT and mediastinoscopy for accurate preoperative staging, allowing them to receive personalized treatment plans that may not be available in general medical units. Therefore, completely rejecting the efficacy of PORT based solely on these 2 RCTs would be irrational, and investigating further is worthwhile. Both trials suffered from a limited number of enrolled individuals and underpowered statistics. Furthermore, participants in the Lung ART RCT were given an outdated radiation therapy that had a higher risk of cardiac toxicity. It seems that for patients with stage IIIA/N0-1, the current findings are consistent with large population-based studies which suggest that using PORT does not provide any survival benefits for resected stage IIIA/N0-1 patients.^[7,11]^ It is worth mentioning that the efficacy of PORT in resected stage IIIB NSCLC patients has seldom been studied before. In our study, we found that resected stage IIIB NSCLC patients would have a better OS after receiving adjuvant radiotherapy.

It was observed in the current study that patients with squamous cell lung cancer, poor histological differentiation grade, and LNR > 1/3 could potentially benefit from PORT. Since treatment options for squamous cell lung cancer are often limited and ineffective, radiotherapy is usually considered as a postoperative treatment option in most cases.^[21]^ Based on the findings of Su et al,^[22]^ postoperative chemoradiotherapy was found to be effective in treating N2-NSCLC patients, especially those with squamous cell lung cancer. Similarly, Tian et al^[23]^ observed that PORT was beneficial for IIIA/N2 NSCLC patients with squamous cell cancer but not for those with lung adenocarcinoma. The LNR is calculated by dividing the number of positive Lymph Nodes by the total number of Lymph Nodes sampled pathologically, and it is a prognostic metric. Urban et al^[24]^ discovered that a high LNR is associated with lower survival rates in patients with resected nodal-positive NSCLC. Additionally, Deng et al^[25]^ used heat maps and prognostic scoring models to estimate the overall survival probability in resected N2 NSCLC patients, and they suggested that LNR could be used as a prognostic indicator for these patients’ survival rates. Wei et al^[26]^ demonstrated that patients with the LNR > 1/3 were more likely to increase the local control rate of tumors after receiving PORT. In Zhu et al^[27]^ study of pN2 NSCLC, patients with sufficient LNR (≥ 30%) drove the majority of PORT OS benefits. In the larger scale study, the results were comparable to those of the aforementioned studies.

There are many independent factors for the prognosis of patients with resected stage III NSCLC. In our study, we regarded that the male, the elderly, patients with squamous cell lung carcinoma, the poor histological differentiation grade carcinoma, patients in stage IIIB and postoperative patients without chemotherapy were associated with worse OS. Wei et al^[26]^ demonstrated that older patients with poorly differentiated tumors were more likely to increase the local control rate of tumors and OS after receiving PORT. This study agreed closely with our results. It is noteworthy that both our univariate analysis and multivariate Cox analysis revealed that the extent of surgery did not influence patient prognosis as an independent risk factor, while postoperative chemotherapy played a significant role. This could be attributed to the benefits of radiotherapy in stage III NSCLC patients, which outweigh any associated risks due to inadequate surgical extent. Our findings are consistent with the results of the subgroup analyses conducted by Arriagada et al^[28]^ and Douillard et al,^[6]^ affirming the therapeutic benefits of postoperative chemotherapy for resected NSCLC patients.

### 4.1. Strength and limitations

Our study revealed that PORT was not universally appropriate for all patients with resected stage III NSCLC. However, a large population-based study indicated that PORT was linked to improved overall survival in patients with resected stage IIIA/N2 and IIIB NSCLC. Moreover, squamous cell lung cancer, poor histological differentiation grade, and LNR >1/3 were more likely to benefit from PORT. In addition, our findings identified that postoperative chemotherapy, rather than the extent of surgery, had a positive effect on patients who underwent PORT.

There are some limitations to this study. Surgical margins, genes, and molecular phenotypes have been reported to be important indicators around PORT for lung cancer; however, these variables were not available in the SEER database, which we used to develop our prognostic model. Additionally, the SEER database did not contain information necessary calculate expanded prognostic outcomes such as disease-free survival, distant metastasis-free survival, and local control rate of tumors in patients with resected stage III NSCLC. Lastly, prospective testing of our model would be necessary to validate our results.

## Acknowledgments

We would like to express our sincere gratitude to Kun Wu, Wang Peng, Zhifeng Shuai, Xin Peng, Haibo Liu, and Sanhong Zhang for their invaluable contributions to this study. We confirm that all individuals have given permission to be named in the Acknowledgments section.

## Author contributions

**Conceptualization:** Kun Wu, Wang Peng, Sanhong Zhang.

**Data curation:** Kun Wu, Wang Peng, Zhifeng Shuai, Xin Peng, Haibo Liu, Sanhong Zhang.

**Funding acquisition:** Sanhong Zhang.

**Investigation:** Zhifeng Shuai, Xin Peng, Haibo Liu.

**Resources:** Kun Wu, Wang Peng, Sanhong Zhang.

**Software:** Kun Wu, Wang Peng, Sanhong Zhang.

**Supervision:** Kun Wu, Wang Peng, Sanhong Zhang.

**Validation:** Kun Wu, Wang Peng, Sanhong Zhang.

**Visualization:** Kun Wu, Wang Peng, Sanhong Zhang.

**Writing – original draft:** Kun Wu, Wang Peng, Sanhong Zhang.

**Writing – review & editing:** Kun Wu, Wang Peng, Sanhong Zhang.
